# Prevalence and Determinants of Burnout Among Palliative Care Clinicians in Saudi Arabia

**DOI:** 10.3389/fpubh.2021.834407

**Published:** 2022-01-20

**Authors:** Eisa Yazeed Ghazwani

**Affiliations:** Department of Family and Community Medicine, College of Medicine, Najran University, Najran, Saudi Arabia

**Keywords:** burnout, palliative care physicians, Saudi Arabia, Maslach Burnout Inventory, burnout-professional

## Abstract

**Background:**

Palliative care is in dire necessity than ever before due to aging populations and the prevalence of cancer and other chronic diseases associated with aging.

**Objective:**

To assess the prevalence and risk factors associated with burnout among palliative care clinicians in Saudi Arabia.

**Methods:**

A cross-sectional study conducted in 2018 covering all palliative care centers of Saudi Arabia and included 44 palliative care physicians (26 males and 18 females). The level of burnout was measured using Maslach Burnout Inventory's (MBI) 22 point scale questionnaire which assesses emotional exhaustion, depersonalization, and reduced personal accomplishment, the three dimensions of burnout syndrome. Pearson correlation and binary logistic analysis were performed using SPSS to find out factors influencing burnout considering *P-*value of <0.05 as significant.

**Results:**

Eight participants (18.2%) had experienced emotional exhaustion and 11 (25%) had experienced depersonalization and detachment, and reduced personal accomplishment, each. Job title and availability of some administrative departments, supporting health care staff, and pain relief medications have shown significant impact of level of burnout. However, the prevalence of the burnout dimensions did not differ significantly according to the palliative care physicians' other characteristics.

**Conclusions:**

This is amongst the first survey to assess the prevalence of burnout among palliative care physicians in Saudi Arabia. Although, some variables have shown significantly high level in the burnout domains, yet, the overall prevalence of burnout is low among palliative care physicians in Saudi Arabia. The availability of hospitals services such as administrative departments, supporting health care staff, and pain relief drugs have shown significant impact on burnout.

## Introduction

As life expectancy is progressively increasing with advances in medical care, the need for palliative care is growing along with the number of patients with incurable and advanced diseases (1).

Hospital-based palliative care provides in a multidisciplinary setup professional physical, psychological, spiritual, and environmental holistic care for patients and their family members aimed at ensuring “death with dignity” (2).

The rising need for palliative care is frequently associated with stressful and demanding challenges for health workers. Since palliative care physicians daily experience suffering and tragedy, they are at risk of burnout (3). Their work entails making difficult ethical decisions and daily encountering patients' distress, dying, and death, which may cause physical, psychological, or work-related stress. If these stressors are not identified and managed on time, palliative care team members may be at risk of burnout (4). Burnout is defined as having three main dimensions: emotional exhaustion (EE), depersonalization and detachment (DD), and reduced personal accomplishment (PA). However, there is no standard definition for burnout yet (5).

Although depression is common with burnout, burnout encompasses a reduced sense of personal accomplishment and reduced self-esteem (6). Burnout is different in that it is specific to the work context rather than encompassing every domain of life. In addition, burnout does not involve physical or biological symptoms (5, 7). However, recent studies and analysis on developments in job-related distress research, as well as ongoing changes in how job-related distress is defined, have revealed some fascinating findings. For instance, while depression can be caused by a variety of causes, a large body of data from well-controlled longitudinal research shows that occupational stressors are linked to higher levels of depressive symptoms (as judged by cause-neutral symptom scales) and a higher risk of depressive disorders. Job stressors are unlikely to increase the risk of burnout without commensurately also increasing the risk of depressive psychiatric illnesses (8, 9).

Burnout may reduce physicians' health care quality and thus a higher potential for medical errors and adverse events. Moreover, burnout has been linked to increased rates of absenteeism and job turnover, reduced productivity and effectiveness at work, as well as patient and staff dissatisfaction (4).

Medicine, particularly palliative care, is regarded as a stressful profession, with the potential for psychological morbidity and burnout. There has been little quantitative research done to document their prevalence among palliative care medical practitioners. This research quantifies the prevalence and risk factors associated with burnout among palliative care clinicians in Saudi Arabia. Several studies have been published on the magnitude of the burnout problem among various medical specialities (10–12). However, there is a paucity of studies on burnout among palliative care physicians (4). Moreover, no previous studies have explored burnout among palliative care physicians in Saudi Arabia to the best of our knowledge. Therefore, this study aimed to assess the prevalence of burnout and its associated risk factors among palliative care physicians in Saudi Arabia.

## Methods

This study employed a cross-sectional design using a simple random sampling technique. It was conducted in 2018 and covered all palliative care centers of Saudi Arabia. During the time of the study, there were 12 palliative care centers located around the Kingdom, six of which have been accredited by the Saudi Commission for Health Specialties. The study population comprised of all palliative care physicians working in Saudi Arabia was (*N* = 51) (13). However, we received 44 completed responses from the participants (response rate 86%). The email addresses of all palliative care physicians were obtained from the Saudi Ministry of Health. An online questionnaire was developed, and all palliative care physicians in Saudi Arabia were contacted through email, briefly informed about the study objectives, invited to participate in the study, and assured complete data confidentiality and anonymity. An informed consent was obtained from all the participants. Ethical approval was obtained from the Scientific Ethical Committee of Najran University. The investigation was carried out in accordance with the principles of Declaration of Helsinki.

### Validity and Reliability of the Arabic-Language Version of Maslach Burnout Inventory (MBI)

The MBI had to be translated into Arabic language because the original version was published in English. This was done by a professional translator. The same was then back-translated and compared to the original version by another professional translator, who found no significant differences. The Arabic-version of the MBI was tested in a pilot study with ten participants for cultural appropriateness, comprehension, and convenience of use. The questionnaire language was found to be understandable and comfortable enough to answer the questions, as well as culturally compatible. The reliability of the study tool was calculated by Cronbach alpha-factor (0.76) and found to be satisfactory. The participants included in the pilot study were included in the final data analysis.

The researcher developed a questionnaire that consisted of the following:

*Demographic and workplace characteristics*: These include age, gender, nationality, medical sector and job title, the availability of supporting staff and services, medications and items related to palliative care, and the average number of patients seen per day.

*Maslach Burnout Inventory*: A MBI version for medical personnel, which includes 22 items across the three dimensions of burnout syndrome, namely emotional exhaustion, depersonalization, and reduced personal accomplishment, was used in this study (14). The respondent is required to answer how often they experience feelings on a 7-point Likert scale that ranges from 0 to 6 (0—never, 1—few times a year, 2—one time a month, 3—few times a month, 4—one time a week, 5—few times a week, and 6—everyday). The questionnaire is reliable, valid, and easy to comprehend, and an Arabic-language version was distributed to the participants.

*Scoring*: Each response was scored for each subscale separately; thus, three scores were computed for each respondent. High scores on the EE and DD and lower scores on PA scale indicates existence of burnout. Scores ≥26 indicate high emotional exhaustion, ≥9 denote high depersonalization, and ≤ 33 indicate reduced personal accomplishment (15).

*Statistical analysis*: The Statistical Package for Social Sciences (SPSS version 23) was used for data entry and analysis. Descriptive statistics (frequency, percentage, mean, and standard deviation) were calculated. The significance of differences was determined using Fisher's exact and likelihood ratio tests instead of the chi-square test, as more than 25% of the expected counts were <5 in most comparisons (16). Binary logistic analysis was performed to predict the factor influencing burnout. Pearson correlation was calculated to find out the association and type of correlation among three dimensions of burnout. Statistically significant differences were considered at *p* < 0.05.

## Results

A total of 44 palliative care physicians completed the study questionnaire, giving a response rate of 88%. The participants' ages ranged from 30 to 59 years (40.9 ± 7.5 years); more than half of the participants (56.8%) were aged 35–45 years, while 20.5% were <35 years old, and 22.7% were >45 years old (Table 1). More than half of the participants (59.1%) were male, while 61.4% were Saudi citizens. About two-thirds (63.6%) had a postgraduate degree in palliative care. Participants' experience in palliative care practice ranged from 1 to 20 years (5.5 ± 4.3 years), with 52.3% having <5 years of experience. Almost half the participants (45.5%) were affiliated with the Ministry of Health, while 31.8% were affiliated with the Ministry of National Guard, 11.4% were associated with the Ministry of Defense, and 6.8% were in the private sector. Consultants constituted 43.2% of the participants; 15.9% were assistant/associate consultants, 9.1% were specialists, 20.5% were residents or general practitioners (GPs), and 11.4% were staff physicians. The average number of patients receiving palliative care seen by more than half of participants (54.5%) was 5–10 patients daily, while 29.5% saw more than 10 patients daily, and 15.9% saw <5 patients daily.

**Table 1:** Personal characteristics of palliative care physicians.

**Personal characteristics**	**Number (*n*)**	**%**
**Age groups(years)**		
• <35	9	20.5
• 35–45	25	56.8
• >45	10	22.7
• Range	30–59		
• Mean±SD	40.9 ± 7.5	
**Gender**		
• Males	26	59.1
• Females	18	40.9
**Nationality**		
• Saudi	27	61.4
• Non-Saudi	17	38.6
Having a postgraduate degree in palliative care	28	63.6
Experience in palliative care practice (years)	28	63.6
• <5	23	52.3
• >5	21	47.7
• Range	1–20	
• Mean±SD	5.5 ± 4.3	
**Medical sector**		
• Ministry of Health	20	45.5
• Ministry of National Guard	14	31.8
• Ministry of Defense	5	11.4
• Private Sector	3	6.8
• Others	2	4.5
**Job title**		
• Consultant	19	43.2
• Assistant/Associate consultant	7	15.9
• Specialist	4	9.1
• Resident/General practitioner	9	20.5
• Staff physician	5	11.4
**No. of palliative care patients/day**		
• <5 patients	7	15.9
• 5–10 patients	24	54.5
• >10 patients	13	29.5

Table 2 shows that 18.2% of participants experienced emotional exhaustion, while one-fourth (25%) of physicians experienced depersonalization, detachment and reduced personal accomplishment. Overall, the prevalence of burnout observed was <25%.

**Table 2:** Dimensions of different burnout scales among palliative care physicians.

**Burnout scales**	**Number (*n*)**	**%**
Emotional exhaustion	8	18.2
Depersonalization and detachment	11	25
Reduced personal accomplishment	11	25

### Relationship Between Participant's Personal Characteristics and Burnout

Table 3 reveals the emotional exhaustion (EE), depersonalization and detachment and personal accomplishment levels among physicians. Job title has shown significant (*p* = 0.004) impact on the emotional exhaustion. No significant impact of other variables is observed. EE was non-significantly higher among the older (9.1%) participants (aged >35 years), holding Post graduate degree in palliative care (11.4%), with <5 years' experience in the field (11.4%) and attending 5–10 patients per day (11.4%). Interestingly, equal percentage of participants demonstrated EE in gender and nationality domain. Similarly, depersonalization and detachment (DD) were non-significantly high among male (15.9%) participants aged 35–45 years (13.6%), Saudis (15.9%), with <5 years' experience in the field (18.2%) and attending 5–10 patients per day (13.6%). A total of 25% of participants have mentioned the reduced feeling of personal accomplishment (PA). Likewise, high level of reduced personal accomplishment was noticed among Saudi nationals (15.9%), middle (35–45 years) age (15.9%) participants, who didn't have post graduate degree (15.9%), having <5 years of experience in palliative care domain (18.2%) and attending 5–10 patients per day (11.4%). We observed the adverse impact (high number of EE, DD and PA) in participants having less (<5 years) experience and attending more patients (5–10/day) on all the three domains of burnout.

**Table 3:** Relationship between participant's personal characteristics with emotional exhaustion, depersonalization and personal accomplishment.

**Personal characteristics**	**Emotional exhaustion**			**Depersonalization and detachment**			**Personal accomplishment**		
	**No**	**Yes**	***P-*value**	**No**	**Yes**	***P-*value**	**Reduced**	**Normal**	***P-*value**
	***N* (%)**	***N* (%)**		***N* (%)**	***N* (%)**		***N* (%)**	***N* (%)**	
**Age groups(years)** ^a^									
• <35	5 (11.4)	4 (9.1)	0.106	6(13.6)	3(6.8)	0.889	3(6.8)	6(13.6)	0.575
• 35–45	23(52.3)	3(6.8)		20(45.5)	6(13.6)		7 (15.9)	19(43.2)	
• >45	8(18.2)	1(2.3)		7 (15.9)	2 (4.5)		1(2.3)	8(18.2)	
**Gender** ^b^									
• Males	22(50)	4 (9.1)	0.697	19(43.2)	7 (15.9)	1.000	6(13.6)	20(45.5)	0.738
• Females	14(31.8)	4 (9.1)		14(31.8)	4 (9.1)		5 (11.4)	13(29.5)	
**Nationality** ^b^									
• Saudi	23(52.3)	4 (9.1)	0.690	20(45.5)	7 (15.9)		7 (15.9)	20(45.5)	1.000
• Non-Saudi	13(29.5)	4 (9.1)		13(29.5)	4 (9.1)		4 (9.1)	13(29.5)	
**Postgraduate degree in palliative care** ^b^							
• No	20(45.5)	3(6.8)	0.448	18(40.9)	5 (11.4)	0.732	7 (15.9)	16(36.4)	0.494
• Yes	16(36.4)	5 (11.4)		15(34.1)	6(13.6)		4 (9.1)	17(38.6)	
**Experience in palliative care practice**									
• <5 years	18(40.9)	5 (11.4)	0.701	15(34.1)	8(18.2)	0.169	8(18.2)	15(34.1)	0.169
• ≥5 years	18(40.9)	3(6.8)		18(40.9)	3(6.8)		3(6.8)	18(40.9)	
**Medical Sector** ^a^									
• Ministry of Health	17(38.6)	3(6.8)	0.873	16(36.4)	4 (9.1)	0.108	6(13.6)	14(31.8)	0.508
• Ministry of National Guard	11(25)	3(6.8)		10(22.7)	4 (9.1)		5 (11.4)	9(20.5)	
• Ministry of Defense	4 (9.1)	1(2.3)		5 (11.4)	Zero		zero	5 (11.4)	
• Private Sector	2 (4.5)	1(2.3)		2 (4.5)	1(2.3)		Zero	3(6.8)	
• Others	2 (4.5)	zero		zero	2 (4.5)		Zero	2 (4.5)	
**No. of palliative care patients/day** ^a^									
• <5 patients	7 (15.9)	Zero	0.582	6(13.6)	1(2.3)	0.804	4 (9.1)	3(6.8)	0.120
• 5–10 patients	19(43.2)	5 (11.4)		18(40.9)	6(13.6)		5 (11.4)	19(43.2)	
• >10 patients	10(22.7)	3(6.8)		9(20.5)	4 (9.1)		2 (4.5)	11(25)	
**Job title** ^a^									
• Consultant	18(40.9)	1(2.3)	0.004^*^	16(36.4)	3(6.8)	0.415	4 (9.1)	15(34.1)	0.7326
• Assistant/Associate consultant	5 (11.4)	2 (4.5)		6(13.6)	1(2.3)		2 (4.5)	5 (11.4)	
• Specialist	1(2.3)	3(6.8)		2 (4.5)	2 (4.5)		Zero	4 (9.1)	
• Resident/GP	9(20.5)	zero		6(13.6)	3(6.8)		3(6.8)	6(13.6)	
• Staff physician	3(6.8)	2 (4.5)		3(6.8)	2 (4.5)		2 (4.5)	3(6.8)	

a *Likelihood ratio test*.

b *Fisher's exact test*.

* *p <0.05*.

### Frequency of Burnout

**Figure 1:**
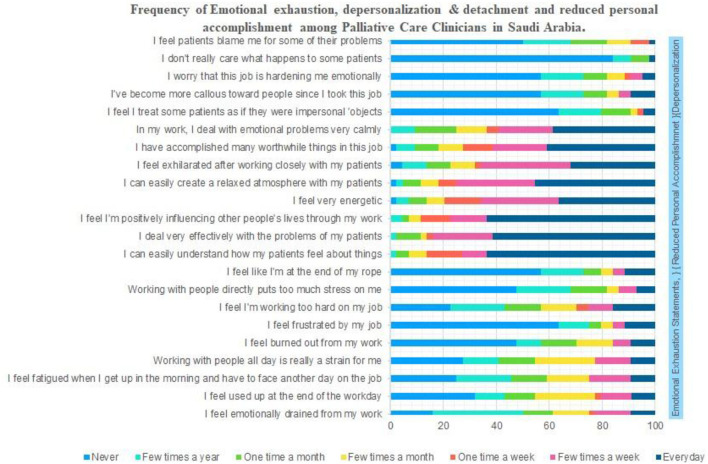
Frequency of emotional exhaustion, depersonalization and detachment and reduced personal accomplishment among palliative care clinicians in Saudi Arabia.

The burnout among palliative care physician was evaluated using 22-point scale (in Figure 1, the bottom 9 statements addressing emotional exhaustion, followed by 8 statements addressing the personal accomplishment and top 5 statement for depersonalization and detachment). Figure 1 depicts the details about frequency of burnout, by considering each statement. Almost 50% of respondents have mentioned that they never experienced any emotional exhaustion during their job or rarely experience it (few times a year). Similarly, more than 50% of physicians have stated, they never get a feeling of detachment and depersonalization regarding their patients. Interestingly, more than 60% of physicians stated that they achieve their feat and accomplishments toward their job and patients that is almost every day or at least few times a week. The similar percentage of participants stated, they never treat their patient as impersonal objects, callous toward their patients, really care for their patients, and their job is not making them emotionally hard (Figure 1).

### Availability of Medical Services and Facilities

**Figure 2:**
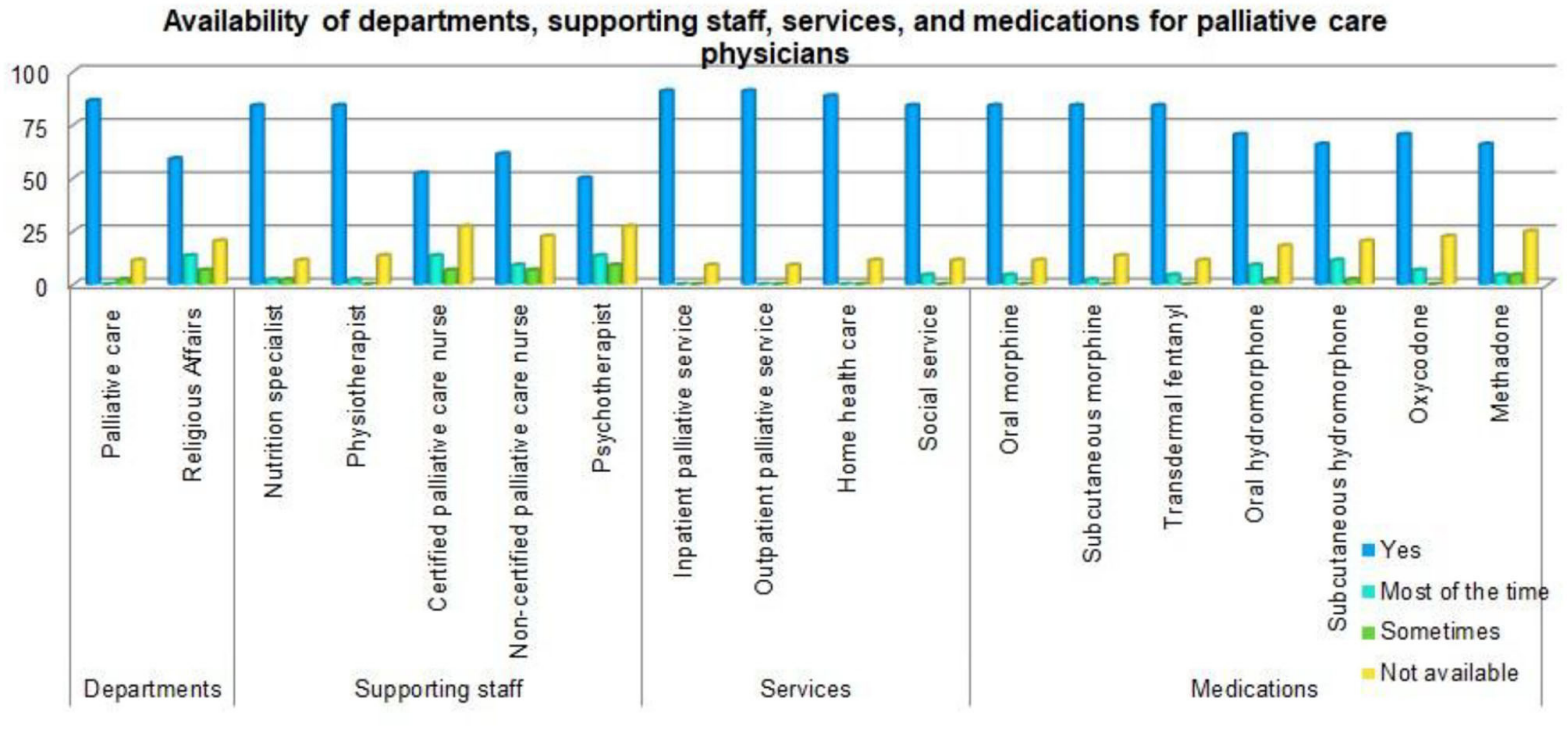
Availability of support services for palliative care physicians in Saudi Arabia.

Figure 2 shows that 11.4% of participants' hospitals did not have a palliative care department, and 20.5% did not have a religious affairs department. Nutrition specialists were not available in 11.4% of hospitals, 13.6% had no physiotherapists, 27.3% had no certified palliative care nurses, 22.7% had no non-certified palliative care nurses, while 27.3% had no psychotherapists. Inpatient and outpatient palliative care services were not available in some hospitals (9.1%), and both home health care and social services were not available in 11.4% of the hospitals. Some hospitals lacked medications, as follows: morphine (oral [11.4%] or subcutaneous [13.6%]); transdermal fentanyl (11.4%); hydromorphone (oral [18.2%] or subcutaneous [20.5%]); oxycodone (22.7%); and methadone (25%).

### Correlation Between Burnout Parameters

Pearson correlation analysis revealed non-significant positive correlation between emotional exhaustion with depersonalization and reduced personal accomplishment, demonstrating that having emotional exhaustion during job will increase the chances of being depersonalized and inferior personal accomplishments. However, negative non-significant correlation was noted between depersonalization and personal accomplishment (Table 4).

**Table 4:** Pearson correlation between emotional exhaustion, depersonalization and detachment and reduced personal accomplishment among palliative care clinicians in Saudi Arabia.

**Variable**	**Emotional exhaustion**		**Depersonalization and detachment**		**Personal accomplishment**	
	***r***	***P-*value**	***r***	***P-*value**	***r***	***P-*value**
Emotional exhaustion	–	–	0.136	0.378	0.136	0.378
Depersonalization and detachment			–	–	−0.030	0.845
Reduced personal accomplishment					–	–

*r, correlation coefficient*.

### Factors Affecting Burnout Among Palliative Care Clinicians in Saudi Arabia

Binary logistic analysis was performed to identify the factors influencing burnout among palliative care physicians. The job title and medical sector had significantly affected the emotional exhaustion (*P* = 0.048) and depersonalization and detachment (*P* = 0.045), respectively. We observed that the risk of emotional exhaustion was 7 times significantly higher among all palliative care physicians (assistant/associate consultants, specialists, resident/general practitioner, and staff physicians) compared to the consultants. Likewise, physicians working in private centers have 5.8 times significant higher risk of depersonalization and detachment compared to physicians working at government palliative care centers. Interestingly, physicians who are aged above 45 years and have experience of more than 5 years in palliative care have 3.2 times higher chance of good personal accomplishment compared to their counterparts (Table 5).

**Table 5:** Factors affecting burnout among palliative care clinicians in Saudi Arabia.

		**Emotional exhaustion**		***P*-value**	**Odds ratio (risk estimate)**		
**Category**	**Subgroup**				**95% confidence level**		
		**Yes *n* (%)**	**No** ***n*** **(%)**		**Value**	**Lower**	**Upper**
Gender	Male	4(9.1)	22(50)				
	Female	4(9.1)	14(31.8)	0.563	1.571	0.337	7.326
Nationality	Saudi	4(9.1)	23(52.3)	0.466	1.769	0.378	8.284
	Non-Saudi	4(9.1)	13(29.5)				
Age	<45 years	7(15.9)	28(63.6)	0.537	0.5	0.053	4.686
	>45 years	1(2.3)	8(18.2)				
Post graduate degree	Yes	5(11.4)	16(36.4)	0.355	2.083	0.431	10.06
	No	3 (6.8)	20(45.5)				
Medical sector	Government	7(15.9)	32(72.7)	0.911	1.143	0.11	11.85
	Private	1(2.3)	4(9.1)				
Job title	Consultant	1(2.3)	18(40.9)	0.048*	7.00	0.78	62.84
	Other	7(15.9)	18(40.9)				
Number of patients seen/day	<10 patients	5(11.4)	26(59.1)	0.586	1.56	0.313	7.77
	>10 patients	3 (6.8)	10(22.7)				
Experience in Palliative care	<5years	5(11.4)	18(40.9)	0.522	0.6	0.124	2.894
	>5years	3 (6.8)	18(40.9)				
		**Personal accomplishment**		***P*** **-value**	**Odds ratio (risk estimate)**		
					**95% confidence interval**		
		**Good** ***n*** **(%)**	**Reduced** ***n*** **(%)**		**Value**	**Lower**	**Upper**
Gender	Male	20(45.5)	6(13.6)				
	Female	13(29.5)	5(11.4)	0.723	0.78	0.197	3.091
Nationality	Saudi	20(45.5)	7(15.9)	0.858	1.138	0.277	4.673
	Non-Saudi	13(29.5)	4(9.1)				
Age	<45 years	25(56.8)	10(22.7)	0.281	3.2	0.353	29.00
	>45 years	8(18.2)	1(2.3)				
Post graduate degree	Yes	17(38.6)	4(9.1)	0.384	1.859	0.456	7.581
	No	16(36.4)	7(15.9)				
Medical sector	Government	28 (63.6)	11(25)	0.17	0.718	0.59	0.874
	Private	5(11.4)	Zero				
Job title	Consultant	15 (34.1)	4(9.1)	0.598	0.686	0.168	2.799
	Other	18(40.9)	7(15.9)				
Number of patients seen/day	<10 patients	22 (50)	9(20.5)	0.34	2.25	0.413	12.24
	>10 patients	11(25)	2(4.5)				
Experience in Palliative care	<5years	15(34.1)	8(18.2)	0.117	3.2	0.719	14.24
	>5years	18(40.9)	3 (6.8)				
		**Depersonalization and detachment**		***P*** **-value**	**Odds ratio (risk estimate)**		
		**Category**	**Subgroup**		**95% confidence interval**		
		**Yes** ***n*** **(%)**	**No** ***n*** **(%)**		**Value**	**Lower**	**Upper**
Gender	Male	7(15.9)	19(43.2)				
	Female	4(9.1)	14(31.8)	0.723	0.776	0.189	3.174
Nationality	Saudi	7(15.9)	20(45.5)	0.858	0.879	0.214	3.612
	Non-Saudi	4(9.1)	13(29.5)				
Age	<45 years	9(20.5)	26(59.1)	0.829	0.825	0.144	4.725
	>45 years	2(4.5)	7(15.9)				
Post graduate degree	Yes	6(13.6)	15(34.1)	0.601	1.44	0.366	5.669
	No	5(11.4)	18(40.9)				
Medical sector	Government	8(18.2)	31(70.5)	0.045*	5.813	0.826	40.884
	Private	3 (6.8)	2(4.5)				
Job title	Consultant	3 (6.8)	16(36.4)	0.219	2.51	0.564	11.161
	Other	8(18.2)	17(38.6)				
Number of patients seen/day	<10 patients	7(15.9)	24(54.5)	0.567	1.52	0.358	6.48
	>10 patients	4(9.1)	9(20.5)				
Experience in Palliative care	<5years	8(18.2)	15(34.1)	0.117	0.313	0.07	1.391
	>5years	3 (6.8)	18(40.9)				

### Impact of Availability of Medical Services and Facilities on Burnout

The impact of availability of hospital services on the overall burnout among clinicians in palliative care section is depicted in Table 6. The results show significant impact of services such as palliative care (*P* = 0.01), religious affairs (*P* = 0.022) and transdermal fentanyl medication (*P* = 0.01) on emotional exhaustion of palliative care clinicians. Similarly, significant impact of nutritional specialist (*P* = 0.048), physiotherapist (*P* = 0.011) and social service (*P* = 0.047) was observed on the level of depersonalization and detachment. Furthermore, availability of home healthcare facility has shown significant (*P* = 0.048) impact on reduced personal accomplishment.

**Table 6:** Impact of availability of medical services and facilities on emotional exhaustion, depersonalization and detachment and personal accomplishment.

		**Emotional exhaustion**			**depersonalization and detachment**			**Personal accomplishment**		
**Facility name**	**Status**	**Yes**	**No**	***P-*value**	**Yes**	**No**	***P-*value**	**Reduced**	**Normal**	***P-*value**
**Departments**										
Palliative care	Available	5(11.4)	34 (77.3)	0.01^*^	9(20.5)	30 (68.2)	0.411	9(20.5)	30 (68.2)	0.411
	Not-available	3(6.8)	2(4.5)		2(4.5)	3(6.8)		2(4.5)	3(6.8)	
Religious Affairs	Available	4 (9.1)	31(70.5)	0.022^*^	8(18.2)	27(61.4)	0.517	8(18.2)	27(61.4)	0.517
	Not-available	4 (9.1)	5(11.4)		3(6.8)	6(13.6)		3(6.8)	6(13.6)	
**Supporting staff**										
Nutrition specialist	Available	6(13.6)	33(75)	0.179	8(18.2)	31(70.5)	0.048^*^	9(20.5)	30 (68.2)	0.411
	Not-available	2(4.5)	3(6.8)		3(6.8)	2(4.5)		2(4.5)	3(6.8)	
Physiotherapist	Available	6(13.6)	32(72.7)	0.3	7(15.9)	31(70.5)	0.011^*^	9(20.5)	29(65.9)	0.612
	Not-available	2(4.5)	4 (9.1)		4 (9.1)	2(4.5)		2(4.5)	4 (9.1)	
Certified palliative care nurse	Available	5(11.4)	27(61.4)	0.473	8(18.2)	24(54.5)	1.000	7(15.9)	25(56.8)	0.434
	Not-available	3(6.8)	9(20.5)		3(6.8)	9(20.5)		4 (9.1)	8(18.2)	
Non-certified palliative care nurse	Available	5(11.4)	29(65.9)	0.270	9(20.5)	25(56.8)	0.678	8(18.2)	26(59.1)	0.678
	Not-available	3(6.8)	7(15.9)		2(4.5)	8(18.2)		3(6.8)	7(15.9)	
Psychotherapist	Available	4 (9.1)	28(63.6)	0.111	7(15.9)	25(56.8)	0.434	7(15.9)	25(56.8)	0.434
	Not-available	4 (9.1)	8(18.2)		4 (9.1)	8(18.2)		4 (9.1)	8(18.2)	
**Services**										
Inpatient palliative service	Available	6(13.6)	34 (77.3)	0.084	9(20.5)	31(70.5)	0.226	9(20.5)	31(70.5)	0.226
	Not-available	2(4.5)	2(4.5)		2(4.5)	2(4.5)		2(4.5)	2(4.5)	
Outpatient palliative service	Available	6(13.6)	34 (77.3)	0.084	9(20.5)	31(70.5)	0.226	9(20.5)	31(70.5)	0.226
	Not-available	2(4.5)	2(4.5)		2(4.5)	2(4.5)		2(4.5)	2(4.5)	
Home health care	Available	6(13.6)	33(75)	0.179	9(20.5)	30 (68.2)	0.411	8(18.2)	31(70.5)	0.048^*^
	Not-available	2(4.5)	3(6.8)		2(4.5)	3(6.8)		3(6.8)	2(4.5)	
Social service	Available	6(13.6)	33(75)	0.179	8(18.2)	31(70.5)	0.047^*^	9(20.5)	30 (68.2)	0.411
	Not-available	2(4.5)	3(6.8)		3(6.8)	2(4.5)		2(4.5)	3(6.8)	
**Medications**										
Oral morphine	Available	6(13.6)	33(75)	0.179	9(20.5)	30 (68.2)	0.411	9(20.5)	30 (68.2)	0.411
	Not-available	2(4.5)	3(6.8)		2(4.5)	3(6.8)		2(4.5)	3(6.8)	
Subcutaneous morphine	Available	6(13.6)	32(72.7)	0.3	8(18.2)	30 (68.2)	0.128	10(22.7)	28(63.6)	0.612
	Not-available	2(4.5)	4 (9.1)		3(6.8)	3(6.8)		5(11.4)	1(2.3)	
Transdermal fentanyl	Available	5(11.4)	34 (77.3)	0.01^*^	9(20.5)	30 (68.2)	0.411	10(22.7)	29(65.9)	0.784
	Not-available	3(6.8)	2(4.5)		2(4.5)	3(6.8)		1(2.3)	4 (9.1)	
Oral hydromorphone	Available	6(13.6)	30 (68.2)	0.58	9(20.5)	27(61.4)	1.000	8(18.2)	28(63.6)	0.367
	Not-available	2(4.5)	6(13.6)		2(4.5)	6(13.6)		3(6.8)	5(11.4)	
Subcutaneous hydromorphone	Available	6(13.6)	29(65.9)	0.725	9(20.5)	26(59.1)	0.829	8(18.2)	27(61.4)	0.517
	Not-available	2(4.5)	7(15.9)		2(4.5)	7(15.9)		3(6.8)	6(13.6)	
Oxycodone	Available	6(13.6)	28(63.6)	0.865	9(20.5)	25(56.8)	0.678	8(18.2)	26(59.1)	0.678
	Not-available	2(4.5)	8(18.2)		2(4.5)	8(18.2)		3(6.8)	7(15.9)	
Methadone	Available	5(11.4)	28(63.6)	0.367	9(20.5)	24(54.5)	0.546	9(20.5)	24(54.5)	0.546
	Not-available	3(6.8)	8(18.2)		2(4.5)	9(20.5)		2(4.5)	9(20.5)	

*Fisher's exact test was applied to find out the p value*.

* *p < 0.05*.

## Discussion

Medicine, particularly palliative care, is regarded as a challenging profession, with the potential for psychological morbidity and burnout. Burnout can be caused by persistent stress and emotionally demanding job requirements for which resources are insufficient. Concerning physicians, emotional exhaustion is defined as a feeling of being “used up” at the end of a workday and having nothing left to offer patients emotionally. Depersonalization manifests itself in feelings of treating patients as objects rather than human beings and a callous attitude toward them (17). Palliative care is in dire necessity than ever before due to aging populations and the prevalence of cancer and other chronic diseases associated with aging (18). Palliative healthcare professionals are constantly confronted with demanding and stressful challenges about providing care with relational and human competencies, taking critical ethical decisions, and daily contact with the suffering, end of life, and death of people for whom they care (17). Such challenges may lead to physical, psychological, and emotional distress and work-related stress, putting palliative care physicians at risk of burnout (19).

In Saudi Arabia, previously, palliative care was not included in the services given by interdisciplinary specialties, and palliative care programs were not integrated into the healthcare system (20). Subsequently, palliative care programs were absorbed within the systems, with an emphasis on hospital organizations, although they were not included as a national standard end-of-life quality indicator (21).

In the year 1992, Saudi Arabia's King Faisal Specialist Hospital and Research Center (Riyadh), established a palliative care center, which progressively proliferated across the country. Although Saudi Arabia has made considerable advances in the field of palliative care, the general public's understanding of end-of-life cancer care and palliation is limited because the focus is on a cure. In 2017, a new end-of-life model of care, “Support me in the last phase of my life,” was established based on systems of care in Saudi Arabia to make end-of-life care more patient-centered (22).

The present study investigated the prevalence of burnout and its associated risk factors among palliative care physicians in Saudi Arabia. The findings reveal that while 18.2% of palliative care physicians experienced emotional exhaustion, a quarter of them experienced depersonalization, detachment, and reduced personal accomplishment.

Parola et al. (3) noted that emotional exhaustion appears when a physician's emotional response is absent. In comparison, depersonalization refers to physicians divesting their characteristics and developing remote approaches to patients and colleagues. This indicates a lack of appreciation for their personal and professional accomplishments and lower motivation and productivity at work.

Dimoska et al. (23) explain that the high prevalence of burnout among palliative care physicians might be related to difficulties with breaking bad news, particularly when it connects to ineffectual management of the disease, add that burnout could be related to a lack of palliative care education and training (23, 24).

The present study's findings reveal the unavailability of some administrative departments (e.g., palliative care and religious affairs departments) and the absence or lack of essential services (e.g., inpatient and outpatient palliative care services, home health care, or social services). Moreover, some hospitals lack a supporting health care team (e.g., nutrition specialists, physiotherapists, certified or non-certified palliative care nurses).

In most low- and middle-income countries, governments have not recognized palliative care as an essential health care service, as exemplified by the frequent unavailability of supporting departments, services, or health care teams for the provision of palliative care and the lack of training for health care professionals and lack of awareness-raising for the general public about the need for and importance of palliative care for cancer patients and other terminally ill patients (25).

Regarding the importance of the religious component of cancer patients' treatment and palliative care, Silbermann et al. (26) argue that a physician's attitude toward palliative care and the devout religious views of many families about the sanctity of life and death usually interfere with the management of cancer patients. They add that religion can further compound end-of-life issues. Muslim families are often skeptical when they hear clear-cut messages about their family member's prognosis from the treating physician. They may be more comfortable receiving information from the religious affairs department and often will respond with, “This is in Allah's hands, and we are not to decide the fate of the patient.” Such attitudes can be attributed to solid Islamic beliefs, including that life expectancy is not to be estimated by the physician but is only up to Allah, who should determine life and death.

In addition, the results show that the essential drugs for pain relief (e.g., fentanyl, hydromorphone, morphine, oxycodone, and methadone) were unavailable in some hospitals.

Silbermann et al. (26) reported that opioids are the most efficient to relieve moderate-to-severe pain in cancer patients. These include natural opiates (e.g., morphine), semi-synthetic opioids (e.g., oxycodone, hydromorphone, hydrocodone), and synthetic opioids (e.g., fentanyl and its analogs). However, Silbermann (27) notes that these pain relief drugs are frequently unavailable to most cancer patients in Middle Eastern countries.

Moreover, Alshammary et al. (21) reported that patients in Saudi Arabia are prescribed much lower doses of drugs for pain relief than in other countries. They attribute this to the shortage of pain relief drugs in Saudi hospitals providing palliative care and highly restrictive opioid analgesic medication policies. Another study (26) noted that the challenge of opioid availability is also due to the lack of training and experience of non-palliative care physicians in safely using such medication.

A high workload, along with a lack of supporting staff, is associated with pressure to meet deadlines, conflicting demands on time, and disruption of home life due to extended work hours. Moreover, having patients with uncontrolled pain, low levels of satisfaction, and inadequate resources for one's role can lead to burnout among palliative care physicians (28, 29).

Regarding the factors associated with burnout among palliative care physicians, the present study shows that emotional exhaustion is significantly higher among specialists, reflecting their increased workload. However, the burnout scales did not differ significantly according to all other personal characteristics. Regarding the risk assessment in the present study, we found that job title and medical sector was a significant risk factor for emotional exhaustion and depersonalization and detachment, respectively.

Maslach et al. (5) reported that people below 40 years of age experience more burnout than those above 40. However, age is confounded with work experience; consequently, physicians may have a greater risk of burnout earlier in their careers. Kamal et al. (30) report that the prevalence of burnout among palliative care physicians is higher among younger clinicians (31). Pereira et al. (24) have observed that more extended experience in palliative care is a protective factor against emotional exhaustion.

Interestingly, dying and death were not considered significant sources of workplace stress among palliative care physicians. There are numerous benefits to practicing palliative medicine that may be protective. These include relationships with patients, perceptions of competence in symptom control, and effectively managing death and dying (30, 32). Furthermore, excessive workloads, inefficient work procedures, clerical burden, lack of input or control, insufficient organizational support, and leadership culture are drivers of the burnout pandemic within healthcare organizations and systems (17).

Workload, control, reward, community, fairness, and values are six aspects of the work environment that are crucial to how clinicians experience work demands, according to Leiter and Maslach (33).

To minimize burnout, Swetz et al. (34) recommend that palliative care physicians be aware of burnout's signs and symptoms to aid early detection and management (35). Moreover, Kaur et al. (2) noted that palliative care providers need an intervention program targeting burnout management. A recent study conducted at the Mayo Clinic in Rochester, Minnesota, USA, found a statistically significant decrease in depersonalization rates when interventional measures were implemented (34). These interventions are directed at individual as well as institutional levels and include team-based interventions, cognitive behavioral therapy, group discussions, mindfulness techniques, professional coaching, mental health training programs, work-hour limitation, and improving the work environment. Comprehensive professional training, such as Cognitive Behavioral Therapy, stress-reduction activities such as mindfulness and group activities, and strict implementation of work-hour limitations for residents are just a few methods that may assist in managing burnout and improve efficiency in health care facilities (36). Increased resilience abilities on an individual level and interventions that change workplace variables on an organizational level are likely to be required for burnout management.

## Conclusions

To conclude, although, some variables have shown significantly high level in the burnout domains, yet, the overall prevalence of burnout is low among palliative care physicians in Saudi Arabia. Some administrative departments and essential services are unavailable in a few hospitals. Moreover, some hospitals lack a supporting health care team, and the essential drugs for pain relief are not available in a few hospitals. Emotional exhaustion is significantly higher among specialists. However, the burnout dimensions do not differ significantly from other personal characteristics.

Therefore, we recommend that palliative care physicians be aware of the signs and symptoms of burnout to promote its early detection and management. There is a pressing need to conduct an intervention program targeting burnout management among palliative care physicians. A sufficient supply of pain relief medications to all facilities providing palliative care services is also crucial. Finally, health care facilities providing palliative care should establish palliative care and religious affairs departments.

## Limitations

The current study was conducted solely among Saudi Arabian palliative care physicians. Hence, the sample size is modest. Apart from that, the participants' self-reporting was used to measure burnout and its related risk factors among palliative care physicians. As a result, data could be skewed due to over-reporting or under-reporting.

## Data Availability Statement

The raw data supporting the conclusions of this article will be made available by the authors, without undue reservation.

## Ethics Statement

The studies involving human participants were reviewed and approved by Scientific Ethical Committee of Najran University. The patients/participants provided their written informed consent to participate in this study.

## Author Contributions

The author confirms being the sole contributor of this work and has approved it for publication.

## Conflict of Interest

The author declares that the research was conducted in the absence of any commercial or financial relationships that could be construed as a potential conflict of interest.

## Publisher's Note

All claims expressed in this article are solely those of the authors and do not necessarily represent those of their affiliated organizations, or those of the publisher, the editors and the reviewers. Any product that may be evaluated in this article, or claim that may be made by its manufacturer, is not guaranteed or endorsed by the publisher.
